# AI Chatbots and Challenges of HIPAA Compliance for AI Developers and Vendors

**DOI:** 10.1017/jme.2024.15

**Published:** 2023

**Authors:** Delaram Rezaeikhonakdar

**Affiliations:** 1. PENN STATE DICKENSON LAW SCHOOL, CARLISLE, PA, USA.

**Keywords:** AI Chatbot, Artificial Intelligence, Law and Medicine, Privacy, Health Policy

## Abstract

Developers and vendors of large language models (“LLMs”) — such as ChatGPT, Google Bard, and Microsoft’s Bing at the forefront—can be subject to Health Insurance Portability and Accountability Act of 1996 (“HIPAA”) when they process protected health information (“PHI”) on behalf of the HIPAA covered entities. In doing so, they become business associates or subcontractors of a business associate under HIPAA.

## 1. Introduction

There are various types of generative AI models, including LLMs. With LLMs being rapidly integrated in the healthcare industry, an increasing number of hospitals, healthcare professionals, and even patients are relying on AI chatbots for various purposes, including workflow optimization.^1^ When an AI chatbot interacts with a user, it initially collects data which is then processed and transformed into a mathematical representation. Subsequently, the chatbot leverages its training data to identify patterns and make predictions regarding the most likely next response of the user or sequence of responses.^2^ The deployment of AI chat bots in the healthcare industry can be accompanied by certain privacy risks both for data subjects and the developers and vendors of these AI-driven tools.^3^

LLMs in the healthcare industry can take different forms. One example is when a HIPAA covered entity — i.e., “a health plan,” “a health care clearinghouse,” or “a health care provider who transmits any health information in electronic form in connection with a transaction covered by” HIPAA^4^ — enters into a business associate agreement with an AI developer or vendor to disclose patients’ electronic medical records.^5^ The AI developer/vendor will be a business associate of the covered entity under HIPAA, and it must comply with HIPAA if it engages in certain activities regarding the PHI on behalf of the covered entity.^6^

Another example is when a hospital or a physician adds input — including patients’ health data — into an AI chat tool to respond to patients’ routine medical questions, medical documentation, generating patient letters, medical summaries, composing emails, improving patients’ understanding about procedures and side effects, and generating clinical and discharge notes, among others.^7^ Furthermore, there can be instances when patients engage in a customized conversation and share their own PHI with an AI chat tool for potential medial questions and recommendations.^8^

Underlying the widespread use and many other potential benefits of generative AI in the healthcare industry, however, certain legal challenges have emerged for AI developers and vendors that expose them to the risk of violating patients’ privacy. This article aims to highlight some of the key measures that AI developers and vendors should implement to effectively manage these privacy risks. In other words, the purpose of this article is to recommend some key strategies to strike a harmonious balance between leveraging the benefits of AI and mitigating its substantial risks. The intended primary group of audience for this article is AI developers and vendors. This article also aims to serve as a valuable resource for policymakers and risk managers as it provides them with relevant information and practical recommendations to effectively manage some of the legal risks associated with AI in the healthcare context.

This article proceeds in five Parts. Part 2 delineates the scope of HIPAA’s protections, explains HIPAA’s safeguards for use, disclosure, and sharing of patients’ PHI with third parties—in this case, AI developers and vendors — and highlights some scenarios of interactions of hospitals, healthcare practitioners, and even patients with AI chat bots where HIPAA does not provide clear guidelines for compliance. Part 3 turns to some of the Federal Trade Commission’s (“FTC”) recent consumer health data and privacy cases — *Flo Health, Easy Healthcare, GoodRX, BetterHelp, 1Health.io*. Part 4 establishes some key takeaways for AI developers and vendors by highlighting the FTC’s increased focus on health data privacy and some risk management considerations. In Part 6, the article will conclude by summarizing its key points.

This article aims to highlight some of the key measures that AI developers and vendors should implement to effectively manage these privacy risks. In other words, the purpose of this article is to recommend some key strategies to strike a harmonious balance between leveraging the benefits of AI and mitigating its substantial risks. The intended primary group of audience for this article is AI developers and vendors. This article also aims to serve as a valuable resource for policymakers and risk managers as it provides them with relevant information and practical recommendations to effectively manage some of the legal risks associated with AI in the healthcare context.

## 2. HIPAA and its Limitations

### 2.1. Scope

HIPAA is one of the leading federal health privacy laws in the United States (“US”). Its primary focus revolves around protection of individuals’ health information, as outlined in the Standards for Privacy of Individually Identifiable Health Information, commonly known as the Privacy Rule.^9^ HIPAA Privacy Rule governs “individually identifiable health information,” referred to as “PHI,”^10^ which is generated by covered entities or business associates.^11^ The term covered entity notably includes “a health care provider who transmits any health information in electronic form in connection with a transaction covered by” HIPAA.^12^ The term business associate refers to a person or organization conducts certain activities on the PHI on behalf of or provide services, such as financial, administrative, management, legal, and data aggregation for a covered entity.^13^

It is noteworthy to mention that de-identified health information, which no longer can be used to identify the data subject, falls outside the definition of PHI, and there is no restriction on use or disclosure of de-identified data under HIPAA.^14^ In other words, as provided by the Department of Health and Human Services (“HHS”), “[h]ealth information that does not identify an individual and with respect to which there is no reasonable basis to believe that the information can be used to identify an individual is not individually identifiable health information.”^15^ De-identification under HIPAA can be achieved through either Expert Determination^16^ (i.e., certification of de-identification by an outside expert) or the Safe Harbor method^17^ (i.e., removal of 18 identifiers including name, dates, city, state, zip code, and age).

### 2.2. Permitted and Prohibited Instances of Data Sharing

Developers and vendors of AI/ML-driven health products require a substantial volume, velocity, variety, and veracity of health information to be able to draw certain patterns in big data.^18^ Protection of PHI under HIPAA from use or disclosure ranges in a spectrum. The highest type of protection offered is when HIPAA requires the covered entity or business associate to obtain the patient’s written authorization to use or disclose a recording^19^ and limits the use or disclosure to the extent “minimum necessary.”^20^ The lowest level of protection is use or disclosure of the PHI without any restrictions.^21^ Furthermore, there are certain situations where disclosure of PHI is mandatory.^22^

**Figure 1:**
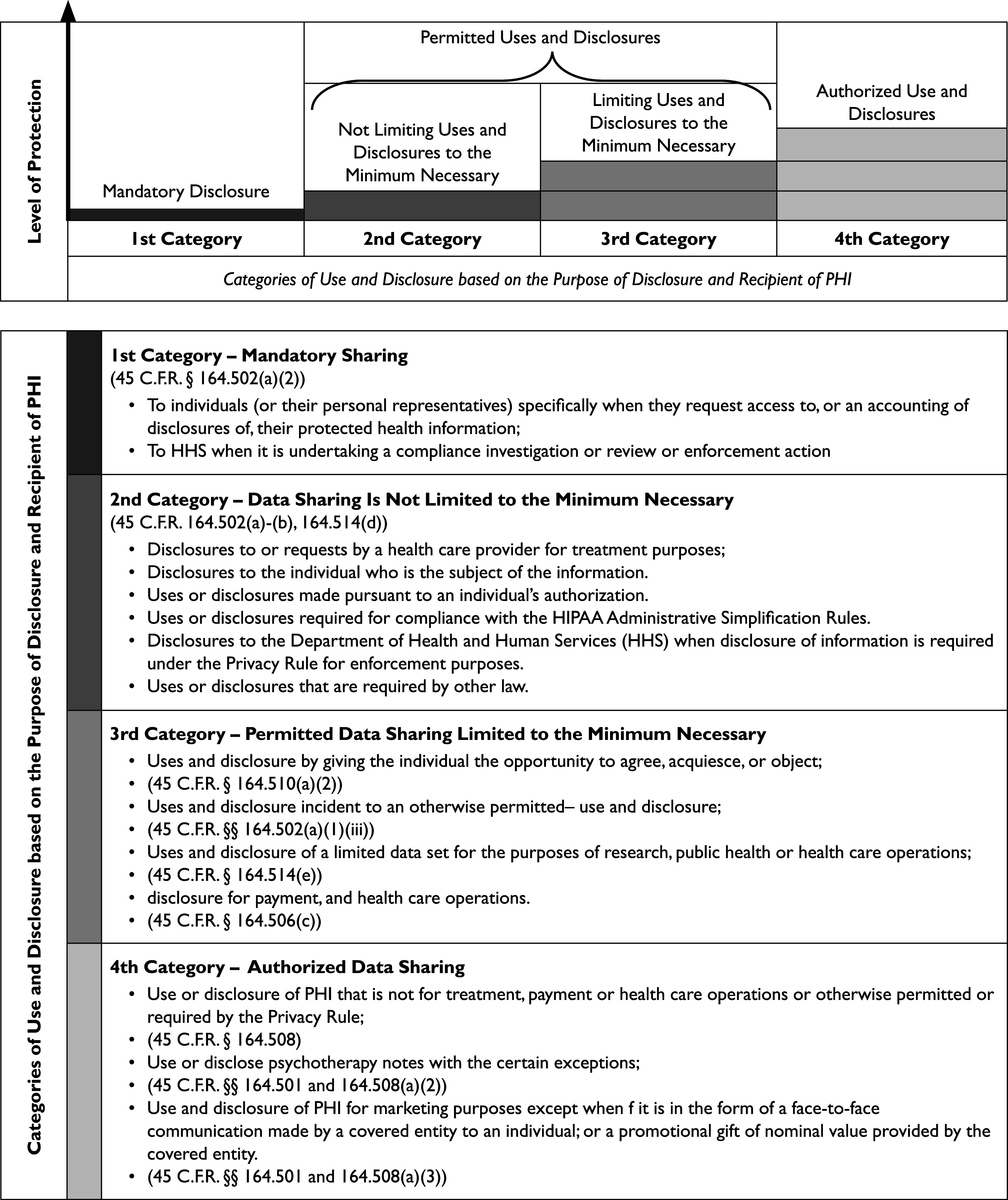
Level of Protection of PHI under HIPAA based on the Category of Data Recipient

The top chart in Figure 1 demonstrates four categories of use and disclosure of PHI under HIPAA. Based on the purposes of use or disclosure, situations when use, disclosure, and sharing of PHI occurs, and type of data recipients, there are four categories as demonstrated by colors red, orange, yellow, and green in both of the charts in Figure 1. Protection of PHI under HIPAA ranges from the lowest level of protection which is situations when a covered entity is obligated to disclose PHI (color red), to when a covered entity may, but is not required, obtain a data subject’s authorization prior to use or disclosure of PHI (and colors orange and yellow), to the highest level of protection which is when a covered entity is required to obtain a data subject’s written authorization prior to use and disclosure of their PHI (color green).

### 2.3. Limitations

There are certain scenarios of AI/ML use in the healthcare industry that HIPAA lacks sufficient protection for patients and clarity regarding the responsibilities of AI developers and vendors.^23^ Also, the Food and Drug Administration (“FDA”) has not provided any guidelines or regulations on LLMs including ChatGPT and Bard either.^24^

The operations of AI developers and vendors on PHI may be left unregulated simply because they do not engage in activities that render them a business associate under HIPAA. When a patient discloses the PHI to an AI chatbot for medical advice, the AI developer or vendor is neither a covered entity, nor a business associate. Similarly, when a hospital or physician discloses patients’ PHI to AI chatbots for various purposes including workflow optimization, that PHI is no longer regulated under HIPAA if the AI developer/vendor is neither a HIPAA-covered entity, a business associate, nor a subcontractor of the business associate.^25^ This is an important deficiency because a considerable number of AI developers and vendors are technology companies that operate outside the traditional scope of HIPAA’s covered entities and business associates framework and thus, patients’ PHI is no longer regulated when processed by these companies.^26^

Furthermore, even if the platform at issue was developed by a covered entity or business associate, the limitation of HIPAA’s scope of regulation implies that if the data subject decided to transfer the PHI to any other spaces, such as a personal health device, that data is no longer protected under HIPAA. The availability of an opt-out option for data subjects using an AI chatbot remains uncertain, as it is not clear whether the AI chatbot users have the same ability to opt out of future data uses as OpenAI users do.^27^

In the given example, the individual is entrusting an advanced platform with their sensitive health information. This platform potentially has the capability to gather a large amount of the user’s personal information from a multitude of available online sources, in most of the cases without the knowledge or consent of data subject.^28^ In this case, the personal information that is not PHI but can be used to draw inferences about data subject’s health information fall outside HIPAA’s purview.^29^ Also, user-generated health information, such as health information posted on social media, despite their sensitivity fall outside the scope of HIPAA.^30^

Last but not least, with massive access of dominant tech companies — such as Meta, Google, and Microsoft — to patients’ personal information, there is a significant risk of privacy violation through re-identification of health datasets that are de-identified through the Safe Harbor mechanism (also known as “data triangulation”).^31^ This concern about re-identification more pronounced when these dominant tech actors integrate generative AI into their own services — For instance, Google integrating chatbot Bard into its search engine or Microsoft integrating ChatGPT-based models into the Office — or when they require the users to rely on their services if they want benefit from the generative AI model — for instance, having to use Microsoft’s Edge browser if an individual wants to use Microsoft’s Bing chatbot.^32^

This issue of data triangulation featured in *Dinerstein v. Google*. ^33^ The plaintiff in that case, Matt Dinerstein, sued defendants, the University of Chicago Medical Center, the University of Chicago, and Google for the invasion of his privacy rights.^34^ Dinerstein stated that sharing his de-identified electronic health records with Google created a significant risk of de-identification due to Google’s access to massive personal information belonging each of its users.^35^

## 3. FTC Act and Health Breach Notification Rule

The FTC has currently taken a proactive stance in protecting health data, thereby intensifying the importance of HIPAA compliance for AI developers and vendors. To protect consumers, the FTC heavily relies on Section 5(a) of the FTC Act and the FTC’s Health Breach Notification Rule^36^ (“HBNR”).

In January 2021, the FTC entered into a settlement with the Flo Health Inc. (“Flo Health”).^37^ Flo Health has developed the Flo Period & Ovulation Tracker — a Direct-To-Consumer (“DTC”) AI-driven health app — that allegedly collected detailed information about menstruations and gynecological health of more than 100 million users since 2016.^38^ According to the allegations of the FTC, contrary to its privacy promises, the company shared consumers personal health information with third parties such as Google, Facebook, Flurry, and AppsFlyer.^39^

Based on the facts of the complaint, the FTC asserted 7 counts against Flo Health: (i) “Privacy Misrepresentation – Disclosures of Health Information”; (ii) “Privacy Misrepresentation – Disclosures Beyond Identifiers;” (iii) “Privacy Misrepresentation – Failure to Limit Third-Party Use;” (iv) Misrepresentation Regarding Notice;” (v) “Misrepresentation Regarding Choice;” (vi) “Misrepresentation Regarding Accountability for Onward Transfers;” (vii) “Misrepresentation Regarding Data Integrity and Purpose Limitation.”^40^

Similarly in May 2023, the FTC filed a complaint against Easy Healthcare Corp. — the developer of the fertility app Premom — for consumer deception, unauthorized data sharing, and failure no notify its users about disclosing their menstrual cycles, reproductive health conditions, and other fertility-related data with third parties — including Google, AppsFlyer Inc. and two China-based firms — for various purposes such as advertising.^41^

Based on the facts of the complaint, the FTC asserted 8 counts: (i) “Privacy Misrepresentation – Disclosures of Health Information;” (ii) “Privacy Misrepresentation – Sharing Data with Third Parties;” (iii) “Deceptive Failure to Disclose – Sharing Geolocation Information with Third Parties;” (iv) “Privacy Misrepresentation – Third Parties’ Use of Shared Data;” (v) “Deceptive Failure to Disclose – Third Parties’ Use of Shared Data;” (vi) “Unfair Privacy and Data Security Practices;” (vii) “Unfair Sharing of Health Information for Advertising Purposes Without Affirmative;” (viii) “Violation of the [HBNR].”^42^

This suit against Easy Healthcare Corp. — was the second attempt of the FTC to hold a company accountable for an alleged violation of HBNR. Only a few months before that, in January 2023, FTC filed a complaint against GoodRX Holdings Inc (“GoodRX”)^43^—a “consumer-focused digital healthcare platform” that “advertises, distributes, and sells health-related products and services directly to consumers, including purported prescription medication discount products.”^44^ Allegedly, the company failed “to notify [more than 55 million] consumers and others of its unauthorized disclosures of consumers’ personal health information to Facebook, Google, and other companies [since 2017].”^45^

Based on the facts of the complaint, the FTC asserted 8 counts: “(i) Privacy Misrepresentation: Disclosure of Health Information to Third Parties;” (ii) “Privacy Misrepresentation: Disclosure of Personal Information to Third Parties;” (iii) “Privacy Misrepresentation: Failure to Limit Third-Party Use of Health Information;” (iv) “Privacy Misrepresentation: Misrepresenting Compliance with the Digital Advertising Alliance Principles;” (v) “Privacy Misrepresentation: HIPAA Compliance;” (vi) “Unfairness: Failure to Implement Measures to Prevent the Unauthorized Disclosure of Health Information;” (vii) “Unfairness: Failure to Provide Notice and Obtain Consent Before Use and Disclosure of Health Information for Advertising;” (viii) “Violation of the Health Breach Notification Rule 16 C.F.R. § 318.”^46^

Following its settlement with GoodRx in February 2023, two other companies went on the FTC’s radar. First, in March 2023, the FTC filed a complaint against BetterHelp Inc (“Better Help”).^47^ The company offered counseling services through its primary website and app, called “BetterHelp,” since 2013.^48^ The FTC alleged that the respondent liable for disclosure of its consumers’ health information for advertising purposes with third parties including Facebook, Snapchat, Pinterest, and Criteo; deceptive privacy misrepresentations; as well as failure to take reasonable measures to safeguard the collected health information.^49^

Based on the facts of the complaint, the FTC asserted 8 counts: “(i) Unfairness – Unfair Privacy Practices;” (ii) “Unfairness – Failure to Obtain Affirmative Express Consent Before Collecting, Using, and Disclosing Consumers’ Health Information;” (iii) Failure to Disclose – Disclosure of Health Information for Advertising and Third Parties’ Own Uses;” (iv) “Failure to Disclose – Use of Health Information for Advertising;” (v) “Privacy Misrepresentation – Disclosure of Health Information for Advertising and Third Parties’ Own Uses;” (vi) “Privacy Misrepresentation – Use of Health Information for Advertising;” (vii) “Privacy Misrepresentation – Disclosure of Health Information; (viii) Privacy Misrepresentation – HIPAA Certification.”^50^

Then, in June 2023, the FTC announced a proposed settlement agreement with 1Health.io Inc. (“1Health”), a provider of DNA health test kits and health, wellness, and ancestry reports.^51^ The FTC argued on several bases that 1Health made misrepresentations about its data privacy practices, including its lack of data deletion processes and a retroactive policy change that enabled genetic data sharing with third parties.^52^

Based on the facts of the complaint, the FTC asserted 5 counts: “Security Misrepresentation - Exceeding Industry Standards;” (ii) “Security Misrepresentation - Storing DNA Results without Identifying Information;” (iii) “Privacy Misrepresentation - Data Deletion;” (iv) “Privacy Misrepresentation - Saliva Sample Destruction;” (v) “Unfair Adoption of Material Retroactive Privacy Policy Changes Regarding Sharing of Consumers’ Sensitive Personal Information with Third Parties.”^53^

**Figure 2:**
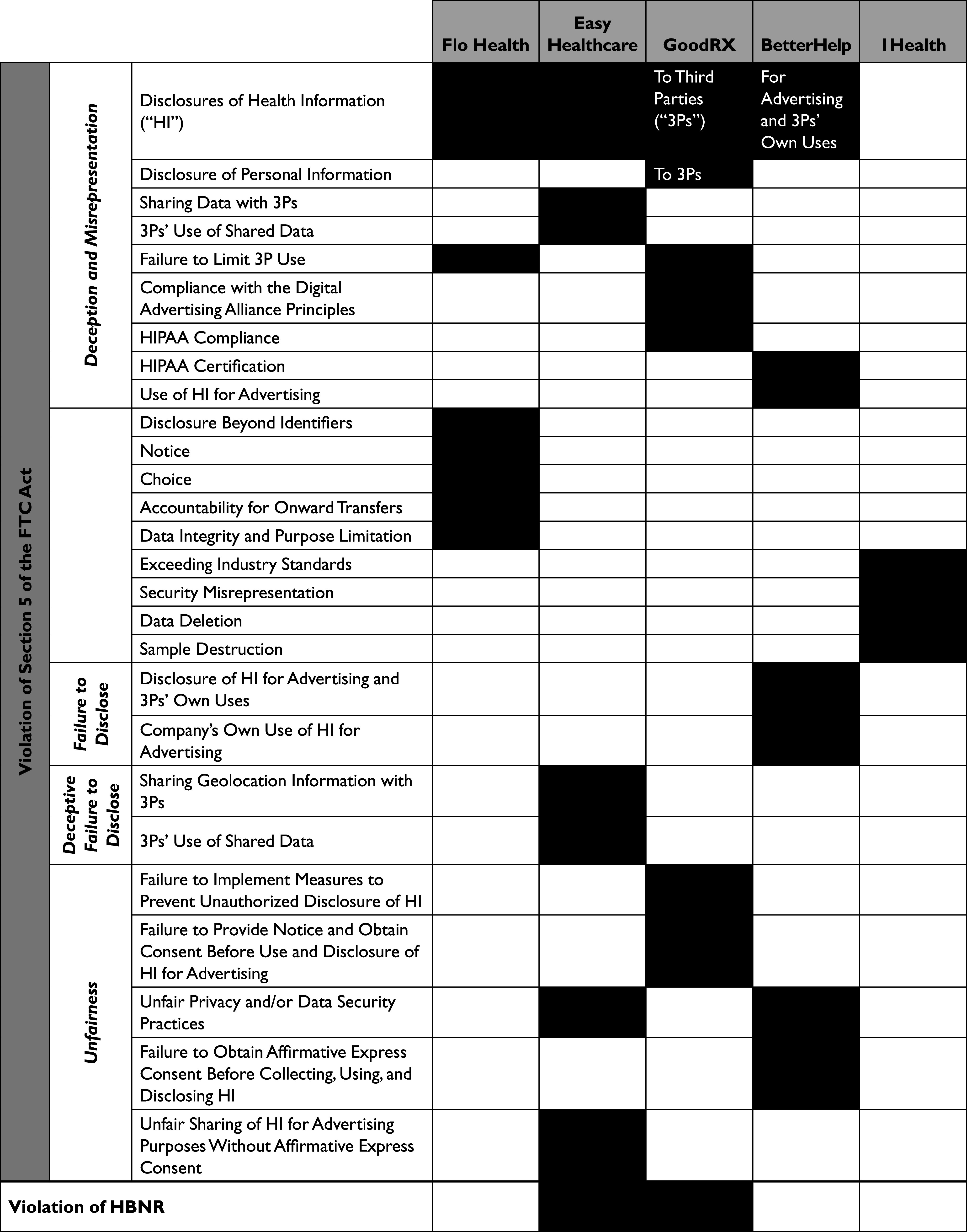
Overview of the FTC Complaints Against Flo Health, Easy Healthcare, GoodRx, BetterHelp, and 1Health

Figure 2 provides an overview of the FTC’s recent consumer health data and privacy cases against the companies that we mentioned in this section. This Figure aims to pinpoint the similarities between these complaints to emphasize the grounds that AI developers and vendors need to be mindful about.

## 4. Considerations for AI Developers and vendors

### 4.1. Guidelines and Enforcement Actions of the FTC

It is true that HIPAA does not provide clear guidelines for compliance. However, AI developers and vendors should treat health data in a way that would be most compliant with not just the letter of HIPAA but with its spirit and purpose. In doing so, they need to take into serious considerations the guidelines and enforcement actions of the FTC that seeks to protect consumers from deceptive or unfair practices or acts in or affecting commerce.^54^

With the increased focus of FTC on health data privacy, collection, use, and disclosure of sensitive health data is very risky, particularly in cases of data sharing with third parties for advertising purposes. To mitigate the potential risks, AI developers and vendors need to be exercise caution, minimize their data collection to what is strictly necessary, and actively engage in monitoring the tracking technologies on their website and apps to prevent any unintended and unlawful collection or sharing of their consumers’ health information. These companies are advised to act with due diligence to notify consumers and obtain their affirmative consent prior to any sort of material changes to their privacy policies such as data sharing for advertising purposes. They should also refrain from any sort of misrepresentation of their privacy compliance or deliberate marketing that causes misunderstanding about the capacities of the offered tool. Lastly, when integrating generative AI into their own services, it is crucial for AI developers and vendors to ensure that this integration aligns with the company’s promises in their privacy policies.

### 4.2. A Risk-Based Approach to Health Data

AI developers and vendors in the digital health space should be mindful of the National Institute of Standards and Technology AI Risk Management Framework^55^ (“AI RMF”) when thinking about how to map, measure, and manage AI risks. The AI RMF is a non-binding framework that was published in January 2023 to facilitate risk management and encourage the trustworthy and responsible use and development of AI systems.^56^ The goal of this framework is “to offer a resource to organizations designing, developing, deploying, or using AI systems [as well as] to help manage the many risks of AI and promote trustworthy and responsible development and use of AI systems.”^57^

AI governance goes hand in hand with data governance. AI developers and vendors are advised to place a primary focus on managing the risks of privacy violations and be diligent to adapt their standards in compliance with new regulations. In addition, to foster trust in AI and reinforcing the company’s commitment to safeguarding consumer privacy in AI applications, AI developers and vendors need to adopt a proactive approach in AI audits and periodically communicate with data subjects about how their data is being handled.

## 5. Conclusion

It is crucial for developers of AI/ML-driven tools to recognize the shortcomings of HIPAA to gain a better understanding about the challenges related to compliance and be mindful about developing appropriate solutions. To achieve this, AI developers and vendors should be familiar with very common scenarios where HIPAA does not extend its coverage to sensitive health data of patients or consumers. This understanding has a critical role in paving the way for addressing these scenarios in a manner that aligns with the policy objectives and the spirit of HIPAA.

AI governance goes hand in hand with data governance, and when combined, allows AI developers and vendors to clearly identify where failures happen within their systems to best protect themselves from potential legal actions as outlined above. In managing compliance risks associated with the collection, use, and disclosure of health data, as well as building trust and credibility with users, AI developers and vendors should avoid any sort of false representations of their privacy policies in any of their open-to-consumers platforms such as their in-app privacy policy or the privacy terms on their website. Furthermore, by diligently assessing AI system’s compliance with legal considerations as well as keeping the users informed about how their data is being handled, AI developers and vendors can foster a privacy-conscious environment.
